# The AI doctor will see you now: assessing the framing of AI in news coverage

**DOI:** 10.1007/s00146-021-01145-9

**Published:** 2022-03-08

**Authors:** Mercedes Bunz, Marco Braghieri

**Affiliations:** 1 Department of Digital Humanities, King’s College London, London, UK

**Keywords:** Agency, Artificial intelligence, Medicine, Healthcare, Framing, Media coverage

## Abstract

One of the sectors for which Artificial Intelligence applications have been considered as exceptionally promising is the healthcare sector. As a public-facing sector, the introduction of AI applications has been subject to extended news coverage. This article conducts a quantitative and qualitative data analysis of English news media articles covering AI systems that allow the automation of tasks that so far needed to be done by a medical expert such as a doctor or a nurse thereby redistributing their agency. We investigated in this article one particular framing of AI systems and their agency: the framing that positions AI systems as (1a) replacing and (1b) outperforming the human medical expert, and in which (2) AI systems are personified and/or addressed as a person. The analysis of our data set consisting of 365 articles written between the years 1980 and 2019 will show that there is a tendency to present AI systems as outperforming human expertise. These findings are important given the central role of news coverage in explaining AI and given the fact that the popular frame of ‘outperforming’ might place AI systems above critique and concern including the Hippocratic oath. Our data also showed that the addressing of an AI system as a person is a trend that has been advanced only recently and is a new development in the public discourse about AI.

## 1 Introduction

‘The AI Doctor Will See You Now’ was the headline of an article in The Wall Street Journal presenting Artificial Intelligence (AI) systems as a person in the role of a doctor (Mims 2018). Similar headlines had been written before (Cohn 2013) and more would follow (Elder 2018; Whitaker 2019; Masters 2020). Those newspaper and magazine articles cover new technological approaches in the healthcare sector based on AI systems. To explain those AI systems to the public, these articles describe the AI systems as outperforming the medical expertise of the doctor taking on her or his role. This is a troubling configuration: describing an AI system as outperforming human expertise is placing it above critique and concern; addressing an AI system as a person replacing the doctor anthropomorphizes the system’s technical functioning. Both framings are sidestepping ethical concerns leaving no space for issues and criticism which Science and Technology Studies (STS) research has shown mediates public involvement. Research experiments have shown that there is a tendency to place exceptionally positive, even god-like expectations on artificial intelligence (Spatola and Urbanska 2020). This article evaluates to what extent this framing, the configuration of AI systems as a person outperforming and replacing the doctor, is common turning to English language news coverage.

## 2 Background

Methods such as *deep learning*, which take advantage of large data sets, have allowed AI systems to profoundly advance, after they gained momentum in 2012 with a paper specifying a new approach (Krizhevsky et al. 2012). By now, a wide range of different AI systems that process images, as well as language, have been implemented to deliver assisting tasks, and reports agree that those systems are projected to impact societies and economies profoundly (Perrault et al. 2019; Crawford et al. 2019). One of the sectors for which these new skills have been considered early on as exceptionally promising is the healthcare sector (Topol 2019). In 2019, the healthcare sector led the global investments into AI with $4 billion (Landi 2020). AI systems applied in this sector automate tasks which so far needed to be done by a medical expert such as a doctor or a nurse, and when AI systems take on these tasks, aspects of agency are being redistributed. While the redistribution of agency can be framed in different ways, it is one particular framing of AI systems, whose configuration will ground our research inquiry in this article: a framing that positions AI systems as (1a) replacing and (1b) outperforming the human medical expert, and in which (2) AI systems are personified and/or addressed as a person. To inquire this, we will examine in parts the English language discourse that explains the arrival of AI systems in healthcare to a general public and analyse to what extent the points 1a, 1b, and 2 are being used in this explanation.

Given that discourses are ‘practices that systematically form the objects of which they speak’, as Foucault (1969, 54) has shown; and given the fact that the introduction of AI systems is heavily being invested in the healthcare sector, we think that it is urgent to draw attention to the role which public discourses in the West assign those AI systems in the area of healthcare. To evaluate this role, we chose to analyse the journalistic discourse of AI healthcare systems in the UK and US through a quantitative and qualitative content analysis of journalistic articles. Our aim hereby is to find and highlight tendencies of framing (Scheufele 1999; Baresch et al. 2010) in some UK and US outlets. We will not deliver a representative result of the overall English language coverage on AI healthcare systems. To evaluate those tendencies, we started by creating a query-based dataset ranging from 1980 to 2019 adopting a geometric progression method in order to highlight relevant case studies for the results ranging from 1980 to 2015, while the results ranging from 2015 to 2019 were run by key collision and nearest neighbour clustering methods on the titles. All entries were subsequently analysed. By analysing journalistic articles using this approach to surface the framing of AI systems and the points 1a, 1b and 2 in its configuration, we situate the research of this article at a crossroad linking aspects of STS research to journalism studies in an interdisciplinary manner. Studies (for example Shoemaker and Reese 2013; Baresch et. al. 2010) using Goffmann’s (1974) concept of framing have shown the news media’s ability to influence audience reception by framing information, which is in our case news covering AI systems in the area of healthcare. Through framing, journalism plays an essential role in configuring our social system and its issues as well as how those issues are presented to a general public. To study how media constructs these issues can effectively be assessed through electronic newspaper databases, as Ridout et al. (2012, 452) state, who name as two reasons for querying journalistic databases to study ‘news media attention to an issue’ and ‘to examine how journalists construct news articles’. In line with these findings, this study examines the news coverage of AI applications in healthcare between 1 January 1980 and 31 October 2019 in a range of English language newspapers from the UK and the U.S. By surfacing this configuration throughout this timeframe, we wish to critically reflect on the framing of AI systems in healthcare in our past and present.

It is important to mention that other configurations describing the agency of AI systems can be found. STS research studying the actual applications of such algorithms in situated settings in bioscience (Lee and Helgeson 2020) or for example also in finance (Mackenzie 2017) have described alternative models of agency such as that of an interoperable collaboration resulting in a distributed agency. The same configuration can also be found when turning to medical perspectives discussing the applications of those algorithms (Topol 2019), an aspect that seems important to mention to show that placing AI systems as an autonomous agency is not unique for the medical discourse. It rather seems that algorithms, which are being described from the perspective of situated settings, appear in a configuration of collaboration. Perspectives on AI systems that are closer to the aspect of innovation, novelty and development, such as tech business or computer science, on the other hand, seem to show a tendency to construct AI systems as an entity outperforming the human expert in the mode of technological solutionism (Morozov 2013), thereby drawing on a much older trope. The tendency to describe technical objects as having ‘a separate, autonomous existence’ (17) has for example already been described by Simondon (2017) in his comprehensive study of the meaning of technical objects, in which he concludes a ‘misunderstanding of the machine’ (16) that can be found throughout Western history (104). In the history of AI systems, this misunderstanding plays a prominent role (Dreyfus 1972; Searle 1980).

Addressing AI systems in a configuration that presents them as an autonomous existence and as a solution outperforming human expertise, however, is of concern. Technical innovations presented as ‘better than the human’ do not leave space for any negotiations, concerns, or issues. How important space for negotiations is, however, has been demonstrated again and again in STS research (Latour 1999; Marres 2007) showing that issues mediate public involvement. Public participation takes place through issues as ‘practices of issue formation are often understood in discursive terms’ as Marres has shown (2007, 761). Furthermore, STS studies into public involvement (Irwin 2001; Wynne 2005) have emphasized that public participation can be constrained by non-participatory issue definitions and that there needs to be an active strive for democratic requirements of inclusivity and accountability. Our research strategy is therefore to look into the discursive configuration of AI systems when it comes to journalistic coverage addressing the public. Our analysis will show that there is a large percentage of articles that construct the AI system as an autonomous system replacing a human’s agency instead of a collaboration assisting, informing, or helping the human thereby leaving no space for issues.

## 3 Method

To build a data set for this study, we chose to use Pro-Quest as a unique data source. ProQuest allowed us to search different news outlets with the keywords ‘artificial intelligence medicine’. The objective was to use keywords that would yield the highest number of results and use the same keywords for all news outlets. We adopted a qualitative approach and chose to focus on three diverse news outlets, The Wall Street Journal, The Daily Telegraph and The Guardian; our corpus is available online divided per outlet. The choice of these news outlets was driven by their diversity, as one focuses on the financial-economic aspect (The Wall Street Journal), one adopts a more conservative stance (The Daily Telegraph) and one adopts a more liberal stance (The Guardian). Our choice is not meant to be representative of the overall news production on the subject, as it is limited to English-language news outlets based either in the United States of America or the United Kingdom. The Wall Street Journal is ‘a business and finance oriented daily title that is the biggest-selling newspaper in the USA and which, since 2007, has been part of the Murdoch empire’ (Harcup 2014). The Daily Telegraph is ‘The UK’s biggest-selling daily quality newspaper, […] Often described as ‘the house organ of the Conservative Party’ (Harcup 2014). The Guardian is ‘A UK-based daily newspaper and online brand owned by a trust rather than a conventional proprietor […] it has retained its role as a liberal voice within a mostly conservative national press’ (Harcup 2014). We applied the same time-frame on all news outlets, ranging from 1 January 1980 to 31 October 2019. The data was downloaded in a CSV file (comma-separated values) which were then uploaded onto and cleaned and profiled with OpenRefine, an Interactive Data Transformation Tool (Verborgh and De Wilde 2013). Data profiling is a process meant to ‘discover the true structure, content and quality’ (Olson 2003, 119) of data. Data cleaning process is a process meant to correct possible errors in the data ‘in a semi-automated way’ (Verborgh and De Wilde 2013, 6).

We have chosen to begin our search on 1 January 1980 as the introduction of computers into ‘patient-management’ in the 1970s was still envisioned ‘as a repugnant interference in what were essentially human activities quite unsuited to any form of mechanisation’ (Payne and Brown 1975, vii). It was not until the end of the 1980s, that the acceptance of computers encountered difficulties within the medical community (Bynum and Porter 2013). Physicians felt ‘threatened by a machine that is superior to man in memorizing facts and retrieving them on command, and now it even aspires to emulate “human” intelligence’ (Anbar and Snell 1986, 298), thus leading to the formal recognition of artificial intelligence as a factor within the realm of medical practice. Applying this timeframe, our corpus comprises 365 results in total.^1^

Overall, The Wall Street Journal search yielded 159 results (44% of the overall data set), The Daily Telegraph search yielded 114 results (31%) and The Guardian search yielded 92 results (25%). The corpus contains the least articles in the decade between 1980 and 1989, 2% of the results (8, all by The Wall Street Journal). The following decades, the results grow continuously: 10% between 1990 and 1999, 22% between 2000–2009, and 65% between 2010–2019 showing that the relationship between artificial intelligence and medicine has attracted a growing amount of journalistic attention since 1980. This corpus lays the ground for further research, which could both diversify the number of news outlets and investigate these news outlets production in terms of subscribers, readers and reactions, for example on social media networks.

## 4 Presentation and discussion of findings

**Fig. 1:**
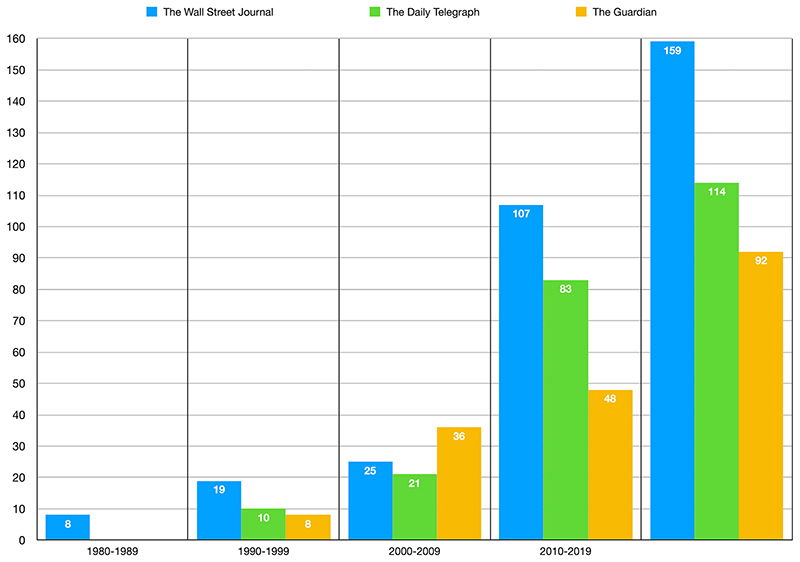
Results for each news outlet (The Wall Street Journal, The Daily Telegraph, The Guardian) per decade

The distribution of yielded search results per year, from 1980 to 2019 (31 October) varies. The Wall Street Journal does not only have the largest number of results but appears to have discussed the theme of AI systems in medicine most consistently. From the end of the 1990s, both The Daily Telegraph and The Guardian started also to cover the subject more consistently with The Guardian leading between 2000 and 2009, before The Wall Street Journal and The Daily Telegraph increased their coverage to catch up. From 2015 onwards we find a quantitative surge, to which The Wall Street Journal and The Daily Telegraph contribute significantly (Fig. 1).

ProQuest provides among other information reference terms for each article entry for each news outlet, selecting the 20 most relevant reference terms (excluding geographical or person-centric reference terms). Whereas the reference term ‘artificial intelligence’ is the most relevant among all three publications, we find that The Wall Street Journal’s reference terms are more cohesive and technically oriented, comprising physicians, algorithms, patients, automation, hospitals, health care, medical research, electronic medical records and medical records among the ten most used. The Daily Telegraph’s ten most used reference terms appear to be more generally adherent to treating artificial intelligence in medicine as a general innovation topic, with employment, researchers, robots, automation, research & development, robotics, innovation, science and social networks among the ten most used reference terms. The Guardian’s twenty most common reference terms are the only ones that comprise specific illnesses or medical practices, such as artificial insemination, cancer, cardiovascular disease, chronic illness, diabetes and family medical history, signalling an attention to the medical aspect of the theme in exam. We will return to this aspect later.

As indicated above, our dataset comprises 365 results. These can be roughly divided in two sections: the results from the years 1980 to 2014 (35 years of coverage) are 169 (46% of the total), whereas results dated from 2015 to 2019 (five years of coverage) are 196 (56% of the total) reflecting the advance in AI systems based on new findings in deep learning. We adopt two different methods to analyse our dataset. For the first period, we sample our dataset in geometric progression for each decade from 1980 to 2014.

**Fig. 2:**
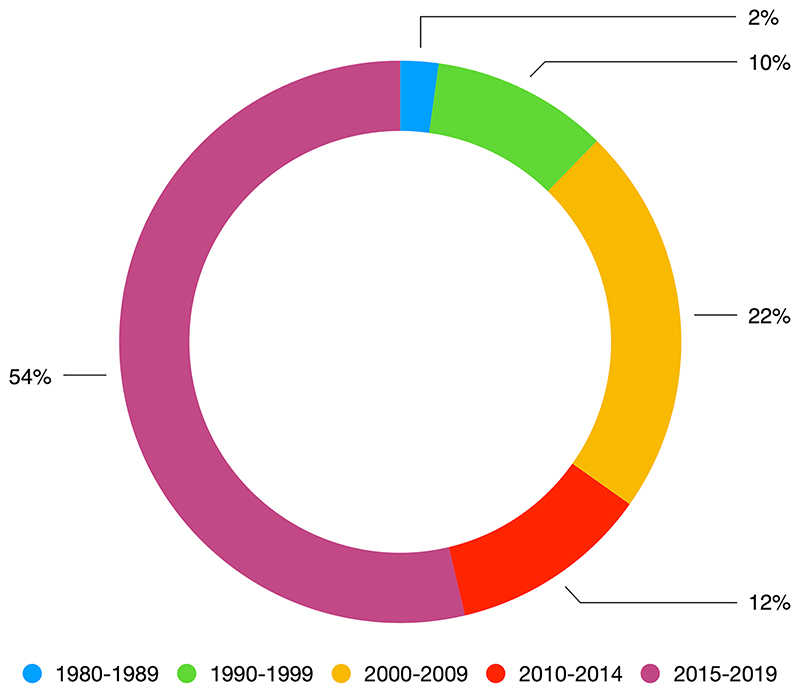
Results per decade and the last 5 years in exam (2015–2019)

Geometric progression is defined as ‘a sequence of numbers in which the ratio of any two consecutive terms is the same’ (Grigorieva 2016, p. 17). Hence, we shall analyse 1 article from 1980 to 1990, 2 articles from 1990 to 2000, 4 articles from 2000 to 2010, 4 articles from 2010 to 2014 (since this period lasts 5 years). From 2015 to October 2019 we shall assess more in-depth the results yielded by our query of the ProQuest database (Fig. 2).

Below, we will discuss the findings for each decade.

### 4.1 Findings from 1980 to 1990, the first decade

In this section, we analyse one sample out of eight results, 12.5% of the total. As we have discussed above, The Wall Street Journal was the only news outlet to yield results for this decade. We found that a significant example of the early coverage of the artificial intelligence and medicine topic is represented by the article ‘Expert-System Software Finds Place in Daily Office Routines’ (Miller 1984). Published in December 1984, the author describes in the article the use of an ‘expert system’ named ‘Puff’ (Pulmonary Function) designed and applied by a lung specialist (Dr. Fallat). The article underlines that ‘expert systems’ such as Puff are ‘much more consistent’ (Miller 1984) than humans in producing a diagnosis:

Some experts may find themselves one-upped by the very systems they design. ‘I’m more likely than Puff to make a human error,’ says Dr. Fallat, the lung specialist. ‘Some days you’re feeling bad and you call everybody severe, whereas if you were feeling better you might have called them mild cases,’ he says. ‘A computer’s much more consistent than that… (Miller 1984).

As we can see, despite the novelty of the application of ‘expert systems’ in the medical field, the ‘computer’ is already depicted as being ‘consistent’. Thus, as early as 1984 we see here the idea of ‘expert systems’ and more broadly computers being able to compete with and outperform human experts. The computer does not bring something new and different to the configuration but is described as something like a human expert—only more consistent. This reflects the approach of the AI community at that time studied by Turkle (2005) or Joerges (1989), who commented: ‘Ever since the invention of “artificial intelligence”, controversies about the “nature” of “thinking machines” have turned around the implicit or explicit question of their likeness to humans’ (Joerges 1989, 32). The depiction of an ‘expert system’ such as ‘Puff’ in the 1984 Wall Street Journal article shows—that the focus of the human likeness is already linked to a competition with the human leading to her or his replacement.

### 4.2 Findings from 1990 to 2000, the second decade

In this section, we analyse two samples out of 37 results, 5.4% of the total. Our second Wall Street Journal article from the following decade is entitled ‘Computer “Brain” Outperforms Doctors in Diagnosing Heart Attack Patients’ (Waldholz 1991). The title literally suggests that artificial intelligence systems in medicine are human-like as they are having a ‘brain’ and presents the computer program in the role of a doctor diagnosing patients:

A computer program outperformed doctors in accurately diagnosing patients with heart attacks in an experiment involving 331 patients complaining of chest pains at a San Diego hospital emergency room (Waldholz 1991).

The information presented in the article draws on the set-up from the research paper ‘Use of an artificial neural network for the diagnosis of myocardial infarction’ (Baxt 1991) in the Annals of Internal Medicine, which presents a higher ‘diagnostic sensitivity’ for the neural network compared to the physicians, while remaining cautious claiming that the AI system ‘may be a valuable aid’. In the editorial published together with Baxt’s study in the Annals of Internal Medicine as well as in The Wall Street Journal article this aid is stressed to act in similar ways as a doctor, whose intuitive expertise is linked to the AI systems’ ‘black box’; or as The Wall Street article puts it:

Drs. Guerriere and Detsky said that while many doctors may be reluctant to trust a “black box,” doctors often rely on intuitive diagnosis made by experts whose ‘thought processes cannot be explicitly passed on to others’. (Waldholz 1991).

Here a parallel is being established between the AI systems black-box and human diagnostic processes, which are further on in the article being equalised.

In this decade, the other two news outlets that we have queried in the ProQuest database start yielding the first results. With The Daily Telegraph, we find a first result in 1992 while the first result for The Guardian shows up seven years later, in 1999. Tim Radford for The Guardian offers a first glimpse into wearable health technology in an article entitled ‘Memory specs and anti-ulcer socks designed for a healthy future’ (Radford 1999). The article reports on research developed by Alice Pentland for the MIT Media Lab:

A short chat with the digital doctor’s assistant about that earache, then it’s off shopping—wearing a wristwatch monitor to keep tabs on pulse and respiration rates, smart socks to detect ulcers, and the ultimate status symbol, memory glasses that mutter ‘you have bought milk and eggs, but you have forgotten the bread’ (Radford 1999).

The idea of a ‘short chat’ indicates a conversation with a person. Here, the futuristic outlook into the future configurates digital technology explicitly in the role of a doctor, which among our samples is the first time. In The Wall Street Journal’s article (Waldholz 1991), the metaphor of the ‘computer brain’ is based on a direct comparison between the AI system and the human leading to the point of outperforming. The framing of the AI systems as a ‘brain’ which refers to a typical discourse in the computer science of AI that gained ground since the 1950s does not discuss the AI system as a person.

### 4.3 Findings from 2000 to 2009, the third decade

In this subchapter, we analyse four samples out of 82 results, 4.8% of the total. The decade between 2001 and 2009 yields 82 results (22% of the total and twice the results of the previous decade) with The Guardian leading with 36 publications followed by The Wall Street Journal with 25 results and The Daily Telegraph with 21 results.

In this decade, we find a certain number of articles which speculate on future applications of robots informed by AI systems for which our sample article in The Daily Telegraph is an example: ‘Automatons for the people: robots are invading our homes, but don’t worry—their intentions are purely benign’ published in 2007. This article clearly configures the robot as a super-nurse that looks after patients and provides them with their medication; it is also able to do the cleaning:

… robots will increasingly find their way into medical centres in the years ahead, not just mopping floors and seeing off superbugs, but also giving out medicine and keeping an eye on patients (Dredge 2007).

While the expertise of nurses seems not something that is relevant to outperform, the tendency to personify the AI is present most clearly in the anthropomorphic expression that the robot is ‘keeping an eye on patients’ (Dredge 2007).

Our sample from The Guardian stems from a special section also looking into the future in its 1 January 2000 edition. The article deviates from our findings so far as it focuses on the limits of artificial intelligence, and cites medicine as one of the fields in which collaboration (rather than a replacement) will be the configuration for a future relationship between artificial intelligence and humans.

… there is still a major obstacle to its application in many fields—it cannot deal with unpredictability. That limits its uses in medicine, for example, and ensures its reliance on some level of human manipulation.’ (The Guardian 2000).

Interestingly, while in this paragraph the AI system is configured as in need of oversight from a human, a different configuration of an AI system is alluded in the headline. Here, we find again the AI system addressed as a robot and configured as a person that will soon take over human jobs and outperform them becoming their ‘boss’. The headline reads: ‘They are not taking over: Robots are not going to boss us. Not yet’ (ibid.).

Our second sample article published in September 2001 in The Daily Telegraph presents news of an account of the first transatlantic operation. Surgeons in New York use remote-controlled robots stationed in a surgery in Strasbourg, France. The news article covering this event describes the situated practice of an AI system assisting the surgeons’ remote movements. Interestingly, the team leader Professor Jacques Marescaux speaks of this experiment as: ‘computer-assisted surgery, where artificial intelligence enhances the safety of the surgeon’s movements during a procedure, rendering them more accurate’ (Highfield 2001). Here, he describes the AI system as a technical system assisting the surgeon with a task. This configuration, however, changes later in the article:

Delicate movements of the surgeon’s hands in New York were transmitted a distance of 4,300 miles to an operating theatre in Strasbourg, where a set of robot arms obeyed their commands. The robot introduced into the women’s abdomen a slender tube called laparoscope, equipped with a tiny, optic-fibre camera, a scalpel and tweezers. The gall bladder was successfully removed in less than an hour and the 68-year-old patient was discharged from Strasbourg Civil Hospital 48 h later (Highfield 2001).

This paragraph shows the fundamental problem with the phenomenon of distributed agency. While in the first sentence, the robot assisted by AI systems is configured as having no agency but as passive—obeying the commands of the surgeons—, tin the following sentence constructs the robot as an actor is introducing the tube into the women; it is not anymore a robot steered by a doctor. The collaboration between human and robot resulting in movements that originate in the doctor but are being corrected by an AI system seems difficult to translate to a broader public.

The rise of robotics informed by Artificial Intelligence systems is also at the centre of a 2004 article published on The Wall Street Journal which focuses on a bionic knee named ‘Rheo’.

Enter the Rheo Knee, a bionic body part that its maker, Ossur, touts as being able to “learn” and adapt to a user’s idiosyncratic movements. “We can basically turn the knee on, have the person walk, and the knee begins to learn how that person walks,” said Scott B. Elliott, a prosthetist with Ossur who has been testing the knee for the past three years. “If they start to change their walking speed, Rheo will be watching” (Zamiska 2004).

While the knee is a solution to a medical problem, the attribution of a name as well as human-like capacities clearly construct the bionic knee informed by AI systems as a person: ‘Rheo will be watching’.

Overall, we find three articles that clearly personify the AI systems reported on, while the fourth one does so only in the headline. In this decade, the trope of outperforming is less strong. The article in The Guardian (2000) that does not personify the AI system. Instead, it constructs the agency of AI as weak and in need of collaboration with a human. However, its headline deviates as it announces that in the future all humans might be outperformed as the robots are ‘taking over’ to become the ‘boss’.

### 4.4 Findings from 2009 to 2014, before the contemporary

In this section, the time period lasts only five years (from 2010 to 2014) instead of a decade. We still continue our geometric progression sampling for the five years between 2010 and 2014 four articles out of our 42 overall results, 9.5% of the total. The first sample is an article published by The Guardian in 2012 devoted to an account of Loebner Prize, ‘which is the oldest Turing Test contest’ (The Society for the Study of Artificial Intelligence and Simulation of Behaviour). It is entitled ‘How far are we from computers that can pass for humans? Quite some way, if a contest for enthusiasts is anything to go by’ (Meltzer 2012). The tone seems now to have become more bolt, which is reflected in the article’s quote by David Levy, an expert for AI systems playing chess, who states:

I think already there are areas of medical diagnosis where it’s been proven that computers can do better than doctors. The problem is there’s a huge amount of litigation. But the logical question is which would you rather be diagnosed by, a human doctor who’s 80% right or a computer doctor that’s 95% right? (Meltzer 2012).

Again, as we observed in an article from the previous decade (Zamiska 2004), the attribution of human-like capacities—or in this case, the outright comparison between a human and a robot doctor—is a narrative element. In our first sample taken from The Wall Street Journal, the effects of using AI systems in healthcare is getting assessed, 30 years after the introduction of ‘complex systems’ such as Puff (Miller 1984). The article written by Nicholas Carr is overall nuanced, although its author addressed the A| system as a threat to human intelligence which clearly comes across in the article’s title: ‘Automation Makes Us Dumb: Human intelligence is withering as we rely more on the artificial variety’ (Carr 2014). The section of the article that is devoted to medicine repeats this threat claiming: ‘The programs incorporate valuable checklists and alerts, but they also make medicine more routinized and formulaic—and distance doctors from their patients’ (Carr 2014). Carr’s article then goes on to cite an academic study blaming the usage of medical software for the misdiagnosis of the first Ebola case in the U.S. by reporting that its authors.

…argue that the digital templates used by the hospital’s clinicians to record patient information probably helped to induce a kind of tunnel vision. “These highly constrained tools,” the researchers write, “are optimized for data capture but at the expense of sacrificing their utility for appropriate triage and diagnosis, leading users to miss the forest for the trees.” Medical software, they write, is no “replacement for basic history-taking, examination skills, and critical thinking.” (Carr 2014).

Interestingly, in this paragraph that is not discussing AI systems but the digitization of medical data, the medical software is not personified. Still, it is addressed as a threat as the software is luring clinicians to apply a ‘tunnel vision’.

Our next sample has been published by The Guardian (Meltzer 2014). This article analyses the role of AI systems on knowledge-based professions, such as law, architecture and medicine. In the section of the article devoted to doctors, Meltzer (2014) interviews extensively Dr Pete Diamandis, the chairman and CEO of XPRIZE. Meltzer’s interviewee introduces a topos, ‘a general argumentative form or pattern’ (Rapp 2010) typical for this decade as the increased focus on ‘Big Data’ precedes that of ‘AI’. This article combines both:

It’s a matter of providing the computer with the data. Once it has the data, it’s able to consider thousands or millions of times more parameters than a human can hold in their head’. […] ‘Eventually, where this is going is that the robot will end up doing the surgeries on its own. I can imagine a day in the future where the patient walks into the hospital and the patient needs, say, cardiac surgery, and the conversation goes something like this: “No, no, no, I do not want that human touching me. I want the robot that’s done it 1,000 times perfectly” (Meltzer 2014).

The argument uses speculative logic as a reinforcer claiming that an AI system can store more information than a human, which is the pretext for an AI system becoming an autonomous entity that can act ‘on its own’. We see here again the aspect of an AI system outperforming the human and that it is linked to the configuration of an AI as a person as the patient wants ‘the robot’ touching him.

Our sample from The Daily Telegraph, an article about Steven Hawking (Williams 2014), takes this aspect of outperformance of doctors one step further and again—like Carr (2014) before—alluding ‘danger’. In it, Hawking describes an AI system developed by Intel that is based on predictive text. This system allows Hawking, who has motor neurone disease, to speak twice as quickly. However, this makes Hawkins worried: ‘The primitive forms of artificial intelligence we already have, proved very useful. But I think the development of full artificial intelligence could spell the end of the human race’ (Williams 2014). Hawking specifies further in the article that ‘technology would eventually become self-aware and ‘supersede’ humanity as it developed faster than biological evolution’ (Williams 2014). Here, the personification of AI is not just a metaphor. Becoming ‘self-aware’ the AI would not just deliver tasks but show typical attributes of being a person such as self-awareness.

In this decade the reporting is becoming more detailed covering a range of different developments advanced by AI systems. However, as AI systems are becoming more and more situated in healthcare, we can also observe next to AI systems outperforming the human a new concern: the AI is threatening the human.

### 4.5 Findings from 2015 to today, the rise of deep learning

In our dataset, articles published between 2015 and 31 October 2019 comprises 196 entries, 56% of the total; among those, entries dated 2015 are 11% of the total, entries dated 2016 are 9%, entries dated 2017 are 22%, entries dated 2018 are 29%, and entries dated in the 10 months we inquired in 2019 are 29% of the total. The coverage of AI systems is clearly increasing. The Wall Street Journal is now again the leading newspaper covering AI systems per entries with 85 (43% of the total), The Daily Telegraph comes second with 74 entries (38% of the total) and The Guardian has 37 entries (19% of the total).

**Fig. 3:**
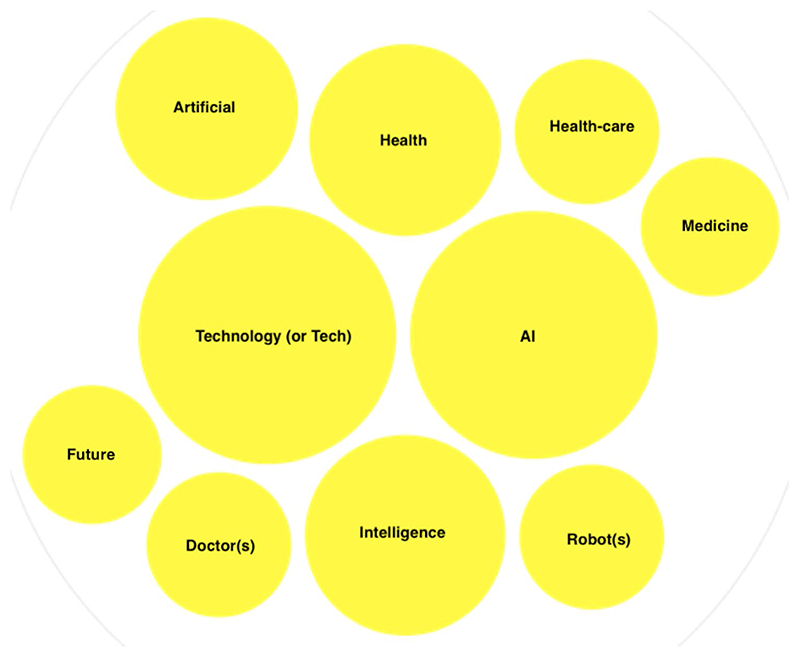
Ten most frequent words in titles (2015–2019). Graph by RawGraph (https://app.rawgraphs.io/)

To provide an overall assessment of all entries, we uploaded our dataset into OpenRefine, an Interactive Data Transformation Tool (Verborgh and De Wilde 2013). Data profiling (Olson 2003) and data cleaning (Verborgh and De Wilde 2013) processes were applied. More specifically, we run Key Collision and Nearest Neighbour Clustering methods on the titles. We applied the key collision method first, followed by the nearest neighbour method. Hence, we were able to assess which are the ten most used words in the 196 titles that comprise our dataset from 2015 to 31 October 2019 (Fig. 3).

The most frequent word in titles between 2015 and 31 October 2019 is ‘technology’ or its variant ‘tech’ with 38 results, followed by ‘AI’ (35 results), ‘intelligence’ (23 results), ‘health’ (21 results), ‘Artificial’ (19), ‘health-care’ (12), ‘robot’ and its plural variant ‘robots’ (12 results), ‘doctor and its plural variant ‘doctors’ (12 results), ‘future’ (11 results) and ‘medicine’ (11 results). Thus, we can observe that within our dataset, the framing of AI systems is technology-related, as we can see from the frequency of use of terms such as ‘technology’ whereas the term ‘medicine’ is approximately 4 times less frequent. Here, it becomes clear that technology and not medicine is playing a bigger role in the configuration of AI systems. This is confirmed by the fact that the term ‘robot’ and ‘doctor’ show the same number of results (12) as well as by the company names being cited in those articles. ProQuest queries provide information on companies cited in the articles, and we assessed which companies and institutions were most often cited within articles. It is due to underline that 121 results out of 196 have at least one company or institution cited, thus this field was empty for 76 results. We erased from our results newspaper companies using the ‘Publication Title’ field. Here, we would like to present the top ten companies cited.

It is relevant to underline that among those top ten companies and institutions cited, Google and its parent company Alphabet is by far the most cited (31 occurrences), almost double the times of the second company, which is Facebook (16 occurrences). Overall, the top ten companies or institutions cited in the dataset are heavily dominated by the tech industry (Google, Facebook, Microsoft and IBM) which represent 68% of the total with 61 references, whereas public institutions (the American Food and Drug Administration, the European Union and the World Health Organization) represent 18% of the total with 17 references, followed by universities (MIT, Oxford University and University of Pennsylvania) which represent 18% of the total with 17 references.

It seems relevant to underline how, within our dataset, artificial intelligence and medicine is a discourse heavily driven by a technological approach, both in the frequency of terms used in titles and in the company and institution references. We believe that it is worth keeping this in mind when discussing the configuration of AI systems as replacing and outperforming the doctor.

## 5 A quantitative and qualitative analysis of the 2015–2019 period

To gain further insight on the database results ranging from 1 January 2015 to 31 October 2019, we decided to implement a new approach. First, we manually cleaned the ProQuest dataset from doubles as some newspapers were using the same article in different editions leaving us with 171 results. Then, we examined each article in the database in order to gather inside into the framing of AI systems assigning a value of 1 if the answer was positive and 0 if the answer was negative^2^ and tested each article’s value in regards to the three following questions.

- Question 1a—replacing the doctor (Q1): In this article, is the AI replacing the doctor?
- Question 1b—outperforming the doctor (Q2): In this article, is the AI outperforming the doctor?
- Question 2—personification of AI (Q3): In this article, is the AI addressed as a person?

**Fig. 4:**
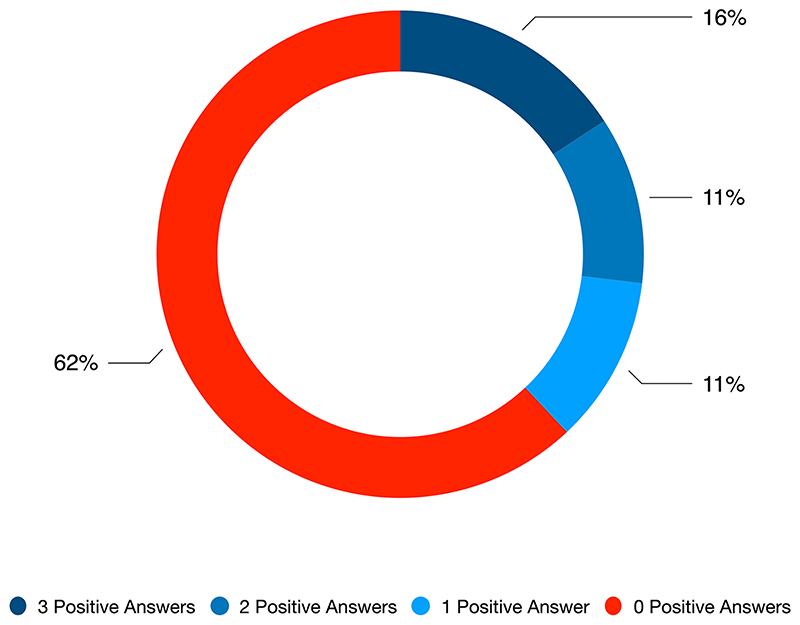
Answers per article to Q1a, Q1b and Q2 in the time period 01/2015–10/2019

As we can see in Fig. 4 below, our query of the ProQuest dataset for the time period 1 January 2015 to 31 October 2019 found 4 articles out of 10 which answered positively to at least one of our research questions, while a slight majority, 62% of results, did not provide positive answers to one of our research questions.

Q1a and Q1b, which mainly revolved around the role of the doctor and its relationship to AI systems yield similar results, with 47 positive answers to Q1a and 49 positive answers to Q1b. The second research question Q2, which revolved around the personification of AI systems (‘In this article is the AI addressed as a person?’) yields a slightly smaller number of positive answers (42). Hence, it is possible to say that within our database the most prominent aspect of the relationship between Artificial Intelligence and medicine regards either the values of replacement or outperformance of practitioners, whereas the personification of AI systems Q2 appears to be less present.

**Fig. 5:**
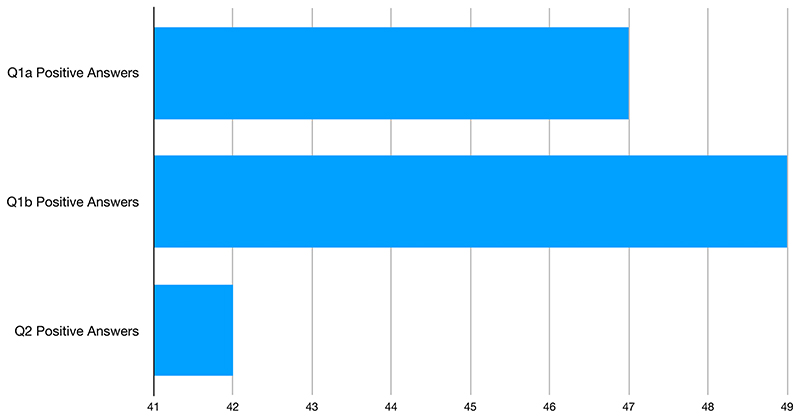
Number of positive answers for each of the research questions

When comparing Q1a, Q1b and Q2 year by year as we can see in Fig. 5, Q1a (replacing the doctor) and Q1b (outperforming the doctor) follow a similar path, peaking in 2018 to fall slightly in 2019, while Q2 (personification of AI) progresses steadily from 2017 onwards and in 2019 overtakes both positive answers to Q1a and Q1b showing that the personification of artificial intelligence within our dataset is clearly evolving.

For this data set, the ‘newsworthy-ness’ of AI systems either replacing or outperforming practitioners seems to peak in 2018, while the personification of AI systems that are being addressed as persons is still increasing further and is a trend in the present depiction of artificial intelligence in medicine picking up a much older trope as this could be found in the analysis of the first section of our dataset, dating from 1980 to 1989 (See Sect. 4.1), with the example ‘expert systems’ such as Puff (Miller 1984), which had not only had a name but was also addressed as ‘more consistent’ than a human.

When turning to the individual newspapers, interestingly in our data set The Wall Street Journal produced with 49% the largest number or articles which answered positively to one or more of our questions Q1a, Q1b, or Q2 with 25% answering positive to all three questions. 51% of articles from The Wall Street Journal in our data set showed no positive values; the Daily Telegraph had 69% and The Guardian 71% of articles with no positive answer in comparison and also yielded smaller positive percentages (both 9%) for answering positive to all three questions.

The Guardian’s dataset is the smallest of the three in exam: 35 entries, compared to 67 for The Daily Telegraph and 73 for The Wall Street Journal. However, it is possible to find a significant example of an article focusing on the possible replacement or outperformance of doctors and AI personification.

In ‘AI equal with human experts in medical diagnosis, study finds’, an article published in 2019, the author starts the article with the introductory sentence: ‘Artificial intelligence is on a par with human experts when it comes to making medical diagnoses based on images, a review has found.’ (Davis 2019). In The Daily Telegraph dataset, the following example from 2015 can be found, entitled: ‘Robot will see you now: AI future of healthcare’ starting with the sentence: ‘Artificial intelligence will bring NHS patients a greater quality of care by better diagnosing medical conditions and personalizing treatment, according to the chairman of NHS England. While our example for The Wall Street Journal is an article from 2019: ‘Paging Doctor AI: Artificial Intelligence promises all sorts of advances for medicine. And all sorts of concerns’, in which Lisa Ward addresses artificial intelligence as a person (Q2), both outperforming (Q1b) and replacing the doctor (Q1a). Artificial intelligence can make diagnoses from digitized images such as mammograms and diabetic retinal scans.[…] Algorithms learn by combing through massive amounts of electronic health records, insurance claims, medical research and other sources. (Ward 2019).

## 6 Conclusions

In our evaluation of the framing of AI systems in healthcare in three English language newspapers from the UK and the U.S. between 1 January 1980 to 31 October 2019, we analysed how often the frame of AI systems as (Q1a) replacing and (Q1b) outperforming the human medical expert, and in which (Q2) AI systems are personified and/or addressed as a person. Our study represents a first, qualitative outlook into the relationship between news media production and the depiction of Artificial Intelligence in the health-care sector.

Our analysis using geometric progression sampling has shown that the personification of AI (Q2) is a frame slowly evolving. While the personification of AI does appear in articles from the first two decades, those articles are being written in the style of science fiction. It could not be found in a general news article. This is only changing slowly. For example, our dataset 2015–2019 reflecting a surge of articles covering AI in healthcare shows no personification of AI for 2015. However, by the last year 2019 the personification of AI systems is the most dominant frame among the three framing approaches we analysed (see Fig. 5). Thus, we can conclude that the personification of AI systems is a more recent trend; one that is by now established as a trope in news coverage.

In the first two decades AI systems are still discussed as a ‘brain’ more than as a person making use of an older trope stemming for computer science (Dreyfus 1972; Searle 1980). The notion of ‘outperforming’ (Q1b), however, is already explicitly alluded to, while the notion of ‘replacing’ remains implicit. In the decade between 2000 and 2009, new notions could be found in our samples. AI informed robots are now having ‘intentions’ (Dredge 2007) and are being considered in the role of a ‘boss’ (The Guardian 2000). With this tendency, elements of ‘replacement’, as well as personification, are starting to evolve. AI systems, even when they are weak AI systems, are now also configured in an active role having agency as well as a name; the sample article which discussed a bionic knee called Rheo alluded that ‘Rheo will be watching’ (Zamiska 2004).

Overall, our research has shown that in our data sample the framing of AI as ‘outperforming’ or ‘replacing’ the doctor, and more recently, the personification of AI systems is a framing that is often used—in our data sample over a third of all articles, 38%, made use of one or more of this framing with 16% using all three values we looked for. News articles covering AI systems from the perspective of tech business such as The Wall Street Journal show a stronger tendency towards the framings we evaluated. Among The Wall Street Journal’s articles between 2015 and 2019, nearly half (49%) were found positive regarding one or more of our frames; 25% of articles showed evidence of all three points: the framing of AI systems as (1a) replacing and (1b) outperforming the human medical expert, as well as (2) addressing AI systems in the role of a person. Interestingly, in our dataset the the reliance on an anthropomorphic frame is much stronger in the financial news coverage than in the other two news outlets. Here, the percentage of articles found positive regarding one or more of our frames is 29% for The Guardian and for The Daily Telegraph 31%. This finding could be explained as follows: coverage focussing on the development of a technology that is still speculative shows a tendency towards anthropomorphisation (2) compared with coverage that reports a more situated setting of concrete technology that is already being tested or used. An interesting point showing potential for further research.

Quantifying the framing of AI systems in healthcare articles allows us to show tendencies when positioning and explaining AI systems [tendencies that can now also be seen beyond healthcare as Curran et. al. (2020) showed]. As those systems become more and more common in healthcare and other sectors, how we present those systems to a general public is essential. By now, different AI systems have already been implemented delivering tasks within healthcare or are being tested, at times supplanting and delivering institutional decisions. By suggesting decisions such as diagnosing an illness or suggesting a personalised treatment, those technologies are taking on societal functions and take part in shaping our societies. And this means that we are in need of space for reflecting on their operation and potential issues. How news articles frame AI systems in healthcare is therefore of central concern—is their framing opening up and inviting a public discussion or closing it down? STS studies (Latour 1999; Irwin 2001; Wynne 2005; Marres 2007) have emphasised that the framing of issues mediate public involvement. Here, an anthropomorphic approach such as the personification of AI may contribute to the creation of wrong assessments. Research regarding the problems of anthropomorphism, which is very advanced in robotics, has shown that the tendency to project human-like capacities onto something can be dangerous, because it profoundly influences people’s evaluation. As Złotowski et. al. (2015, 356) have shown, when evaluating AI or robots people tend to ‘have much higher expectations regarding their capabilities’. Likewise, Pedersen and Johansen (2020: 523) have argued that ‘whereas the inherent traits of simple tools are easily decoded, the same traits are not easily decoded—nor even discovered—in advanced technology’ such as AI systems. Now that those systems are being introduced to assist with healthcare, however, we need an open debate about their ‘traits’ and to encourage an understanding of their technical capacities and difficulties (Thomas 2020).

The politics of healthcare delivery needs to remain democratic, an aspect reflected in the fact that doctors are being bound to ethical values such as the Hippocratic oath. With this article, we wished to provide factual numbers to show that there is a tendency to present AI systems as outperforming human expertise, which is placing it above critique and concern and beyond the Hippocratic oath; and that the rising trend of addressing an AI system as a person replacing the doctor is an anthropomorphisation of the system’s technical functioning, which may be covering up potential ethical issues.

## Data Availability

The dataset which is used in this article has been uploaded onto the Open Science Framework website at: https://osf.io/qhvtu/. The dataset is publicly available and has a DOI (10.17605/OSF.IO/QHVTU).

## Footnotes

1 The dataset which is used in this article has been uploaded onto the Open Science Framework website at: https://osf.io/qhvtu/.

2 The dataset which is used in this article has been uploaded onto the Open Science Framework website at: https://osf.io/qhvtu/.
